# Behavioral modeling and neuroimaging of impaired risky decision making in patients with chronic musculoskeletal pain

**DOI:** 10.1117/1.NPh.10.2.020901

**Published:** 2023-05-18

**Authors:** Xinglin Zeng, Wen Tang, Fei Gao, Ziyue Tang, Zhou Zhang, Juan Zhang, Meng Du, Zhiyi Chen, Xueli Chen, Zhen Yuan

**Affiliations:** a University of Macau, Centre for Cognitive and Brain Sciences, Macau, China; b University of Macau, Faculty of Health Sciences, Macau, China; c The First Affiliated Hospital, Sun Yat-Sen University, Department of Rehabilitation Medicine, Guangzhou, China; d Fudan University, Institute of Modern Languages and Linguistics, Shanghai, China; e University of South China, Institute of Medical Imaging, Hengyang Medical School, Hengyang, China; f Xidian University, School of Life Science and Technology, Xi’an, China

**Keywords:** chronic musculoskeletal pain, risky decision-making, computational modeling, functional near-infrared spectroscopy, prefrontal cortex

## Abstract

**Significance:**

Performance during risky decision making is one of the essential cognitive functions that is impaired in several psychiatric disorders including addiction. However, the cognitive mechanism and neural correlates underlying risky decision making in chronic pain patients are unclear. To our knowledge, this study is among the first to construct computational models to detect the underlying cognitive process of chronic pain patients during risky decision making.

**Aim:**

This study aimed at inspecting the significantly abnormal risky decision-making patterns of chronic pain patients and its neuro-cognitive correlates.

**Approach:**

In this case-control study, 19 chronic pain patients and 32 healthy controls (HCs) were included to measure the risky decision making in a balloon analogue risk task (BART). Optical neuroimaging using functional near-infrared spectroscopy, together with computational modeling, was carried out to systematically characterize the specific impairments based on BART.

**Results:**

Computational modeling findings on behavioral performance demonstrated that the chronic pain patient group exhibited significant deficits in learning during BART (p<0.001), tending to make decisions more randomly without deliberation (p<0.01). In addition, significant brain deactivation alternation in the prefrontal cortex (PFC) during the task was detected for the patient group compared with that from the control group (p<0.005).

**Conclusions:**

Long-term aberrant pain responses significantly disrupted the PFC function and behavioral performance in chronic pain patients. The joint behavioral modeling and neuroimaging techniques open a new avenue for fully understanding the cognitive impairment and brain dysfunction of risky decision making associated with chronic pain.

## 1 Introduction

Pain affects over 50 million people in the United States, with ∼30.7% of US adults suffering from chronic pain.^1^ In particular, chronic pain has become a common yet complex and challenging issue around the world, threatening public health and increasing the economic burdens of governments.^2^ Chronic pain is denoted as the pain lasting for over three months, which is substantially associated with emotional distress and functional disability.^3^ More importantly, chronic musculoskeletal pain, as the most predominant among all chronic pain conditions, represents a severe challenge to primary care. In addition, previous studies^4^ demonstrated that 10.4% to 14.3% of chronic pain patients suffered from moderate to severe disabling pain. Further, patients with severe and intractable chronic pain might develop psychiatric disorders, such as depression and anxiety, leading to psychological distress, job loss, or social isolation.^5^ Therefore, the complicated nature of chronic pain involves an interplay between psychological and physical factors, causing increased emotional distress and reduced quality of life.^6^^,^^7^

Interestingly, neuroimaging studies have revealed the altered brain activities of afferent and descending pain pathways (e.g., thalamus, insula, and somatosensory cortex) and the atrophy of different pain perception regions of the brain after experiencing long-lasting abnormal pain stimulus. In addition to the brain regions associated with pain pathways, regions of emotional response, including amygdala and nucleus accumbens, have also been observed to exhibit abnormalities in cases of chronic pain, in line with the findings from neuropsychiatric disorders.^8^ Specifically, those altered brain regions are responsible for corresponding cognitive functions. Several cognitive deficits have been identified in chronic pain, such as memory decline^9^ and attention impairments.^10^ However, as an integral part of human higher-order cognition, risky decision making is poorly understood among chronic pain patients. To date, no studies have been performed to inspect the neural correlates of risky decision making for chronic pain patients.

Decision making constitutes an integral part of daily life. It involves a complex process whereby individuals try to fulfill their aims and expectations based on their judgments regarding rewards and risks under internal and contextual/social constraints.^11^ Previous studies have illustrated that impaired decision making is one of the core features of several neuropsychiatric disorders, such as depression^12^ and anxiety.^13^ Additionally, individuals’ performance and potential neural correlates in decision making are also used for the detection and treatment assessment of mental disorders.^14^ Similarly, decision-making behaviors can also be negatively influenced by an abnormal pain stimulus^15^^,^^16^ in which disrupted decision making was detected as an attempt to offset the negative experience of pain and acute thermal pain. In addition, the relationship between risky decision-making performance and pain stimulus was also inspected,^17^ demonstrating that the experience of pain or the threat of additional pain might cause changes in risky decision making and efforts on other cognitive tasks. More importantly, it was discovered that chronic pain patients exhibited cognitive deficits in attention and working memory, which are crucial for the behavioral performance of risky decision making.^18^

In particular, the balloon analogue risk task (BART) has been widely used to measure risk-taking behaviors. BART is able to illustrate a real-world situation to identify an overall propensity for risk taking rather than a unique likelihood of engaging in a particular type of risky behavior.^19^ Meanwhile, BART was reported to be the only behavioral instrument that was unaffected by recall bias.^20^ The adjusted BART scores, defined as the average number of pumping for unexploded balloons, are used to measure the degree of risk taking.^21^ However, the adjusted BART scores are unable to reveal some nuanced information, including learning ability and loss aversion. To address this issue, computational modeling of cognitive tasks has been widely used in various clinical patients to overcome the limitations of conventional behavioral tasks.^22^ Specifically, three models have been proposed to fit the BART behaviors. Initially, Wallsten et al.^23^ proposed a four-parameter model to depict the behavior in BART; then van Ravenzwaaij, Dutilh, and Wagenmakers^24^ proposed a two-parameter model by removing two parameters from the four-parameter model, which showed a better fit. Recently, Park et al.^25^ proposed a new computational model by utilizing the prospect theory, which showed significantly improved performance over the previous work. Until now, no studies have systematically examined the specific cognitive impairments of chronic pain patients relevant to BART. It is therefore important to quantitatively depict the underlying cognitive process of BART by constructing computational models to inspect the specific deficiency in chronic pain patients. Yet, the two-parameter approach has a major limitation, assuming that participants do not learn during BART,^24^ which mitigates the explanatory power of modeling. Thus, considering the learning process of BART, we used the other two models to recover the performance of BART,^23^^,^^25^ which provided viable tools for estimating the specific deficit in risky decision making among the chronic pain population in the current study.

In addition, to investigate the neural correlates of risky decision making among chronic pain patients, functional near-infrared spectroscopy (fNIRS) neuroimaging was carried out. As a noninvasive optical neuroimaging technique, fNIRS measures the concentration changes of oxygenated hemoglobin (HbO) and deoxygenated hemoglobin (HbR) in brain tissue in response to neuronal activation. This technique has several distinctive advantages, including compatibility with other devices, low cost, robustness against motion artifacts, higher temporal resolution compared with functional magnetic resonance images, and higher spatial resolution compared with EEG.^26^ Drawing on fNIRS technique, previous studies demonstrated the altered structure and functions of prefrontal cortex (PFC) for chronic pain patients. For instance, Donadel et al.^27^ demonstrated distinct activation patterns at PFC elicited by pain stimulus between chronic pain patients and HCs using fNIRS. Meanwhile, after effective intervention for chronic pain patients, significantly reduced activations in PFC were detected during pain stimulus.^28^ Furthermore, abnormal PFC activity was also identified during reward processing^29^ and attention tasks^30^ in chronic pain. In light of this existing evidence, it is hypothesized that chronic pain patients would manifest different brain activation patterns in PFC than that of the HCs, as revealed by fNIRS data.

Therefore, this study aims to inspect the cognitive impairment associated with risky decision-making in patients with chronic musculoskeletal pain. It is hypothesized that risky decision making was significantly impaired in chronic musculoskeletal pain patients compared with that from the HCs. To test the hypothesis, concurrent behavioral modeling and fNIRS neuroimaging were performed to inspect the specific impairments and potential neural mechanisms during BART. In particular, using the computational model, nuanced cognitive processes during BART would be revealed, which could further relate to the abnormalities that contribute to the aberrant BART performance. Meanwhile, fNIRS recordings can capture the significant difference in brain activation between chronic pain patients and HCs. It is expected that this study might provide new insights into the cognitive neural mechanism associated with risky decision making in chronic musculoskeletal pain.

## 2 Methods

### 2.1 Participants

Nineteen chronic musculoskeletal pain patients (25.3±4.6 years, 12 females, Table 1) who reported chronic pain lasting over 6 months, with visual analog scale (VAS) ratings of less than 7, participated in this study. All patients were diagnosed with chronic musculoskeletal pain and received non-medication treatment at the First Affiliated Hospital of Sun Yat-sen University (Guangdong, China). Thirty-two age- and gender-matched adults (24.7±4.2 years, 18 females) were recruited as the HCs. Both patients and HCs were right-handed and reported no history of neurological or psychiatric disorders (including depression and anxiety) or insomnia. Informed consent forms were obtained from all participants before the experiment. The protocol and all procedures of this study were approved by the Institutional Review Board from Sun Yat-sen University and the University of Macau.

**Table 1:** Demographic and chronic pain characteristics of the patient group.

Participant number	Gender	Age	Pain condition	Pain duration
1	Male	27	Chronic neck pain	More than 6 months
2	Male	21	Chronic neck pain with hands numbness	More than 1 year
3	Female	26	Dizziness with soreness of neck muscles	More than 6 months
4	Female	30	Suboccipital muscles pain	More than 1 year
5	Female	22	Neck pain	More than 2 years
6	Male	20	Neck pain with dizziness	More than 1 year
7	Female	28	Left shoulder pain	More than 9 months
8	Male	28	Hip pain	More than 1 year
9	Female	24	Knee pain	More than 1 year
10	Female	31	Neck pain	More than 6 months
11	Female	28	Shoulder pain	More than 2 years
12	Female	27	Shoulder pain	More than 4 years
13	Male	26	Neck pain	More than 6 months
14	Female	29	Knee osteoarthritis	More than 1 year
15	Male	28	Shoulder pain	More than 1 year
16	Female	19	Left shoulder pain	More than 6 months
17	Male	20	Low back pain	More than 1 year
18	Female	32	Recurrent myofascial pain	More than 1 year
19	Female	15	Left knee pain	More than 6 months

### 2.2 Paradigm and Procedures

**Fig. 1:**
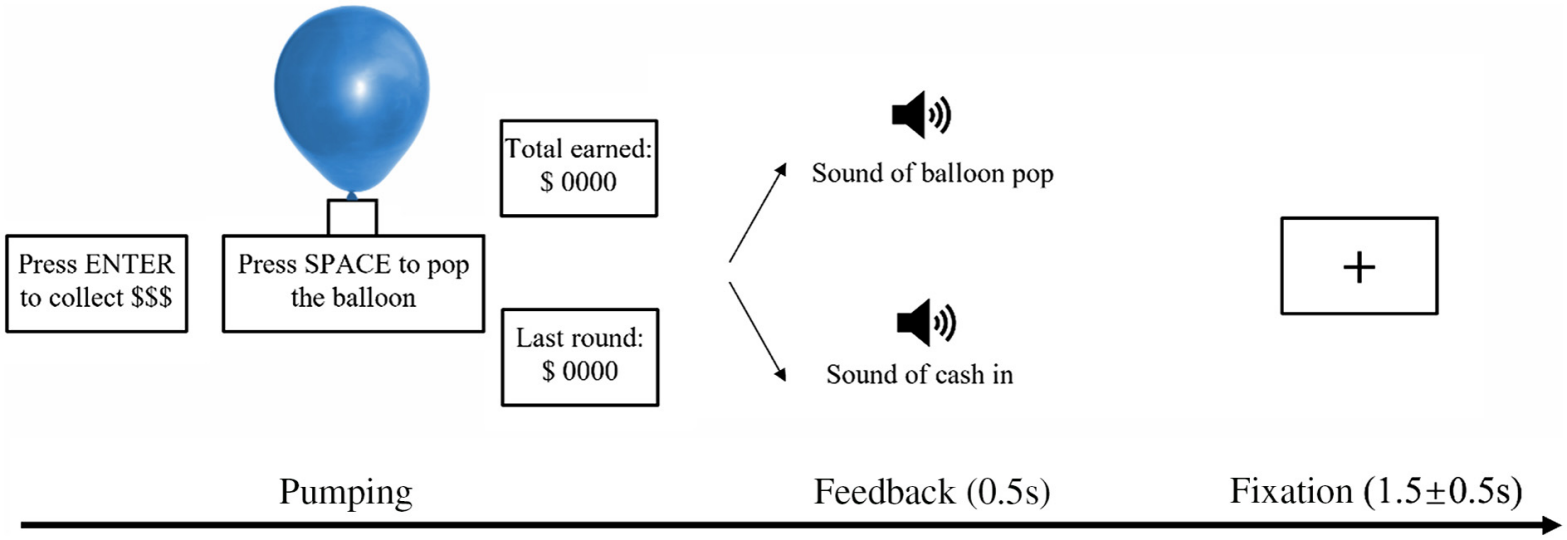
Schematic of the modified BART. The trial of BART consisted of the pumping period, feedback period (sound of the results, 0.5 s), and recovery period (a fixation cross lasting for 1 to 2 s).

BART is composed of pumping, feedback, and recovery periods. At the beginning of the pumping period (Fig. 1), a virtual balloon with one of the three colors (blue, green, and red) was presented in the center of the screen, and participants were instructed to decide whether to pump the balloon to continually enlarge the predescribed rewards or alternatively to terminate this trial to receive the rewards that have been received on this trial by pressing the corresponding buttons. The pumping period would be ended immediately either by the participants’ choice to stop, winning the collected rewards, or when the balloon exploded itself and participants lost all of the collected rewards on the trial. In addition, the different colors of the balloon were associated with differing maximum pumping times: 8 pumping times for the blue balloons, 32 for the green, and 128 for the red. Meanwhile, the time point of pumping explosion for each trial was randomly determined. Therefore, participants were able to perform the experiment with different sets of explosion probability, such that they were unaware of the probability of balloon explosion. Feedback comes with the sound of a balloon pop or cash out, and the recovery period lasts for 1 to 2 s with a fixation cross displayed in the center of the monitor.

Participants were seated in a quiet and dark room, and both behavioral data and fNIRS signals were recorded simultaneously during BART. Before the experiment started, the participants were required to obtain as much reward as possible from the task, which was associated with their monetary reward at the end. After an 8-trial practice session, participants would perform the formal test consisting of 90 trials of 3 conditions (i.e., 3 different balloon colors). It took 8 to 12 min to finish the tests, and the reward was offered to each participant based on his or her performance in the task.

### 2.3 Computational Modeling

As mentioned earlier, two computational models were chosen based on existing studies,^23^^,^^25^ which showed an instinctive interpretation of the learning process. Both computational models used the number of pumps and the outcome of pumps (explosion or not) in BART.

#### 2.3.1 Re-parameterized four-parameter model

The re-parameterized four-parameter model (RFPM) was based on two crucial assumptions: (i) participants need to renew their belief about the balloon’s explosion probability after each trial and (ii) participants can determine the optimal number of pumps before each trial. According to previous reports,^23^ the behavioral patterns of BART were determined by four factors: prior belief of the balloon not bursting (ϕ), learning rates based on the results of each trial (η), risk-taking propensity (γ), and inverse temperature (τ) quantifying the degree of determination of choices.

Based on the first assumption, $p_{k}^{\text{burst}}$ is a constant probability that participants believe a pumping would result in the balloon explosion in trial k. Then participants update prior beliefs after the feedback on each trial, which is denoted as

$$p_{k}^{\text{burst}}=1-\frac{\phi +\eta \sum_{i=0}^{k-1}n_{i}^{\text{success}}}{1+\eta \sum_{i=0}^{k-1}n_{i}^{\text{pumps}}}\;\text{with}\;0<\phi <1,\;\eta >0,$$ (1)

in which $\eta \overset{k-1}{\underset{i=0}{\sum }}n_{i}^{\text{success}}$ refers to the sum of successful pumps up to trial k−1 and $\eta \overset{k-1}{\underset{i=0}{\sum }}n_{i}^{\text{pumps}}$ denotes the sum of all pumps until trial k−1.

As for the other assumption, participants have to determine the optimal pump number ($v_{k}$) prior to each trial, without adjustment during BART. Therefore, the likelihood that participants can pump balloon on trial k for pump l, $p_{kl}^{\text{pump}}$ is written as

$$v_{k}=\frac{-\gamma }{\ln(1-p_{k}^{\text{burst}})}\;\text{with}\;\gamma \geq 0,$$ (2)

$$p_{kl}^{\text{pump}}=\frac{1}{1+e^{\tau (l-v_{k})}}\;\text{with}\;\tau \geq 0.$$ (3)

The likelihood of the data p(D|α,μ,τ,γ) depends on the probability that participants can pump on trial k for pump l, $p_{\mathrm{kl}}^{\text{pump}}$. The probability of the abovementioned parameters is basically the product of all probabilities with one minus the probability of cash out conditions.

$$p(D|\alpha ,\mu ,\tau ,\gamma )=\overset{k^{\text{last}}}{\underset{k=1}{\prod }}\overset{l_{k}^{\text{last}}}{\underset{l=1}{\prod }}p_{kl}^{\text{pump}}{\left(1-p_{k,l_{k}^{ast}+1}^{\text{pump}}\right)}^{d_{k}}.$$ (4)

#### 2.3.2 Exponential-weight mean-variance model

Park et al.^25^ proposed the exponential-weight mean-variance model (EWMVM) to precisely depict the performance of BART, considering some crucial attributes of risk taking, including loss aversion and impulsive responses. Five factors were adopted to clearly describe the behavioral patterns of BART: prior belief of the balloon not bursting (ϕ), learning rates based on the results of each trial (η), risk-taking propensity (γ), inverse temperature (τ), and loss aversion (λ).

In particular, a weight was proposed for prior belief (ϕ), which is determined by the learning rate (η). Therefore, Eq. (1) is rewritten as

$$p_{k}^{\text{burst}}=\frac{1}{1+\eta \sum_{i=0}^{k-1}n_{i}^{\text{pumps}}}*(1-\phi )+(1-\frac{1}{1+\eta \sum_{i=0}^{k-1}n_{i}^{\text{pumps}}})*P_{k-1}\;\text{with}\;0<\phi <1,\eta >0,$$ (5)

in which $P_{k-1}$ denotes the observed probability in trial k−1 and $\frac{1}{1+\eta \sum_{i=0}^{k-1}n_{i}^{\text{pumps}}}$ measures the weight for prior belief (ϕ).

In addition, Park et al.^25^ applied mean-variance analysis to appraise subjective utilities ($U_{kl}^{\text{pump}}$) for balloon pump on trial k for pump l, which is calculated as

$$U_{kl}^{\text{pump}}=(1-p_{k}^{\text{burst}})r-(1-p_{k}^{\text{burst}})\lambda (l-1)r+\gamma p_{k}^{\text{burst}}(1-p_{k}^{\text{burst}}){\left\{r+\lambda (l-1)r\right\}}^{2}\;\text{with}\;\lambda >0,$$ (6)

in which r represents the number of coins for each successful pump and $p_{\mathrm{kl}}^{\text{pump}}$ is calculated by subjective utilities ($U_{kl}^{\text{pump}}$) as

$$p_{kl}^{\text{pump}}=\frac{1}{1+e^{\tau (0-U_{kl}^{\text{pump}})}}\;\text{with}\;\tau \geq 0.$$ (7)

Similarly, in p(D|α,μ,τ,γ,λ), the five parameters are estimated using the same method as Eq. (4).

#### 2.3.3 Parameter estimation

Parameters of the two models were accessed using hierarchical Bayesian analysis,^31^ which allows for calculating the individual and group data simultaneously in a mutually constraining fashion. Hierarchical Bayesian analysis was available in the Stan software package^32^ and hBayesDM package^33^ in R^34^ using Hamiltonian Monte Carlo. A large sample size of 4000 was used to ensure that the parameters were able to be converged to the goal distributions, and four independent chains were adopted to inspect whether the posterior distributions were independent of the initial starting points.

#### 2.3.4 Model comparison

The performances of the two models were compared and justified using leave-one-out information criterion (LOOIC).^35^ LOOIC was calculated from the leave-one-out cross-validation, estimating out-of-sample prediction accuracy based on the log-likelihood captured from the posterior distributions. A low LOOIC score represents a superior model performance. LOOIC weight is defined by Akaike weights^36^ based on LOOIC values as

$$w_{i}(\mathrm{LOOIC})=\frac{\exp\{-\frac{1}{2}\Delta_{i}({\mathrm{LOOIC}}_{i}-{\mathrm{LOOIC}}_{\min})\}}{\sum_{n=1}^{N}\;\exp\{-\frac{1}{2}\Delta_{n}({\mathrm{LOOIC}}_{i}-{\mathrm{LOOIC}}_{\min})\}}.$$ (8)

### 2.4 fNIRS Data Acquisition and Preprocessing

**Fig. 2:**
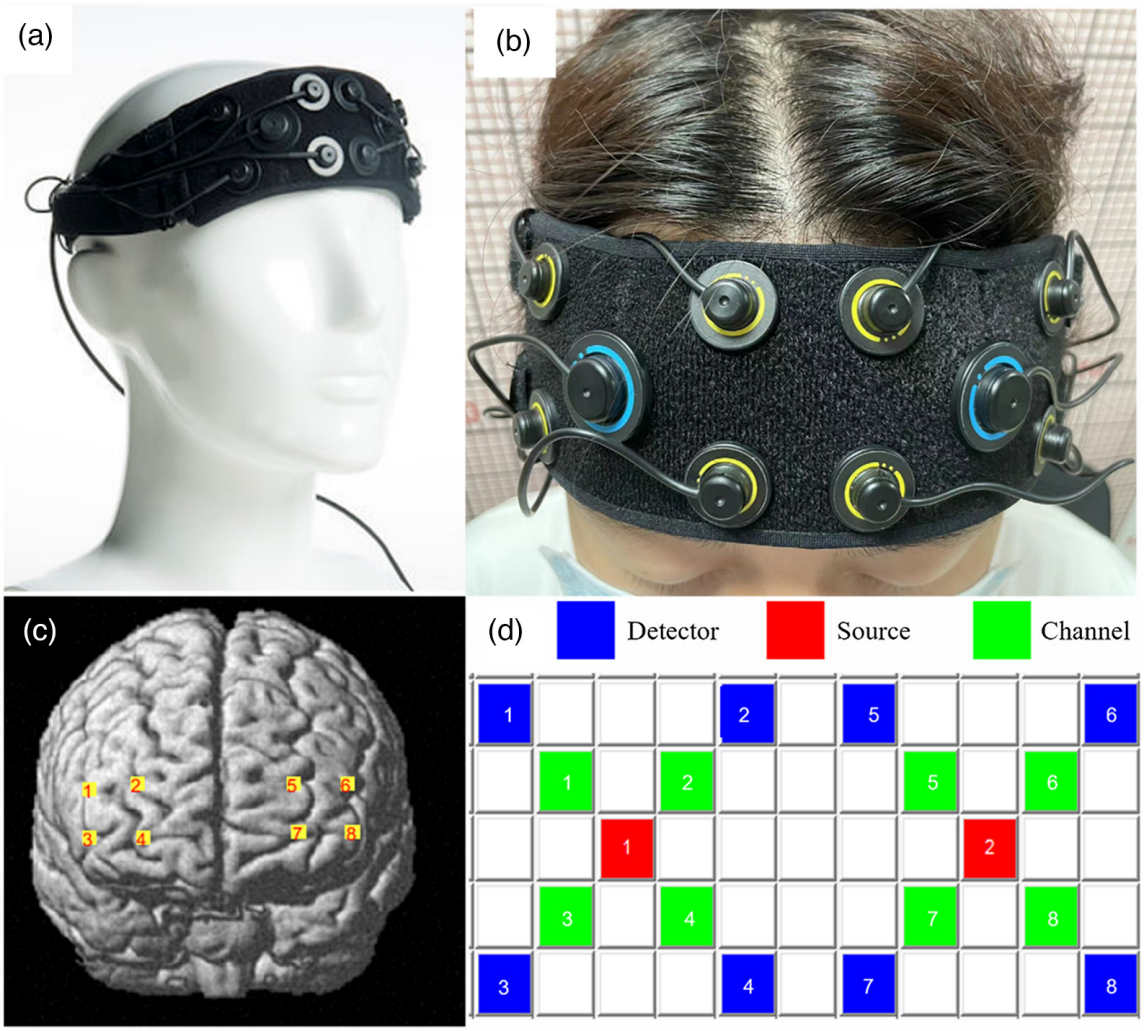
Artinis device and channel position. (a) The Artinis device, composed of two near-infrared light source emitters and eight detectors; (b) the device setup; (c) fNIRS channels reconstructed by NIRS-SPM; and (d) fNIRS topomaps.

The Artinis device (Oxymon Mk III, The Netherlands) was utilized to measure the HbO and HbR changes in PFC with a sampling rate of 50 Hz at two wavelengths of 760 and 850 nm. Specifically, two near-infrared light sources and eight detectors with an inter-optode distance of three cm yielded eight fNIRS channels. In addition, the Montreal Neurological Institute (MNI) coordinates^37^ of each fNIRS channel were quantified using the ICBM-152 head model, which were then processed by NIRS-SPM for spatial registration to estimate the anatomical labels and the overlapping percentage of covered brain regions (Table 2, Fig. 2).

**Table 2:** 3D MNI coordinates, anatomical labels, and coverage percentage of fNIRS channels.

Channel number	MNI			Brodmann Area - anatomical label	Percentage of overlap
	X	Y	Z		
1	51	46.33	16.33	45 - Pars triangularis Broca’s area	0.60517
				46 - Dorsolateral PFC	0.39483
2	31.67	65	17	10 - Frontopolar area	0.80989
				46 - Dorsolateral PFC	0.19011
3	50.33	51.67	−1.33	45 - Pars triangularis Broca’s area	0.029197
				46 - Dorsolateral PFC	0.9635
				47 - Inferior prefrontal gyrus	0.0072993
4	30.67	68.33	−1.67	10 - Frontopolar area	0.36093
				11 - Orbitofrontal area	0.63907
5	−25.67	66.67	17.67	10 - Frontopolar area	0.86716
				46 - Dorsolateral PFC	0.13284
6	−46	49.33	17.33	45 - Pars triangularis Broca’s area	0.41985
				46 - Dorsolateral PFC	0.58015
7	−27.67	67.33	0.67	10 - Frontopolar area	0.51495
				11 - Orbitofrontal area	0.48505
8	−47.67	51.67	−0.67	10 - Frontopolar area	0.022642
				45 - Pars triangularis Broca’s area	0.037736
				46 - Dorsolateral PFC	0.93962

fNIRS data were preprocessed using NIRS-KIT,^38^ implemented in MATLAB. Recordings from the first and last 15 s were removed from each participant’s raw data to ensure that the participant could keep a steady state. Then, the detrend^39^ and temporal derivative distribution repair methods^40^ were adopted to reduce the data drift and correct artificial motions, respectively. To reduce the potential physiological noise and pursue a satisfactory signal-to-noise ratio, the data were further filtered with a bandpass of 0.01 to 0.1 Hz.^41^

To estimate the brain activation in the PFC during the risky decision-making task, a general linear model (GLM) at the individual level was utilized.^39^ For the GLM model, the task condition for risky decision making was convolved with a standard canonical hemodynamic response function to form the corresponding regressor, and the resting data (45 s) were included as the implicit baseline. Then, the individual activation of decision making (β: beta value) for each measurement channel was generated and contrasted with the baseline PFC activity. Although the HbO signals are more sensitive to detecting the regional cerebral blood flow changes in cognitive tasks, only HbO signals were included for further analysis.^42^^,^^43^

### 2.5 Statistical Analysis

In accordance with a case-control study protocol, the current statistical analyses were done in R software 3.6 and MATLAB 2016. First, a two-way ANOVA revealed that the BART scores were significantly different (F(2,49)=47.821, $p<2\times 10^{-16}$) among the three balloons, whereas no significant difference of BART scores across the three conditions between chronic pain patients and HCs (F(2,49)=1.013, p=0.316) and interaction effects were found (F(3,48)=1.05, p=0.726). Given that the difference between three conditions was huge regarding maximum pumping times and the BART scores, the adjusted BART scores were compared by independent sample t-tests (two-tailed) across the three conditions, respectively. Then, the two models were compared by LOOIC to select the better fit. Model parameters, including prior belief of the balloon not bursting (ϕ), learning rates based on the results in each trial (η), risk-taking propensity (γ), inverse temperature (τ), and loss aversion (λ), were measured by independent sample t-tests (two-tailed) to explore the difference between the chronic pain patients and HCs. For those analyses, the significance level was set at 0.05, and p-values of fNIRS results were corrected using the false discovery rate (FDR). Due to the preliminary nature of the work, no *a priori* power analysis was conducted.

## 3 Results

### 3.1 Performance of BART

**Fig. 3:**
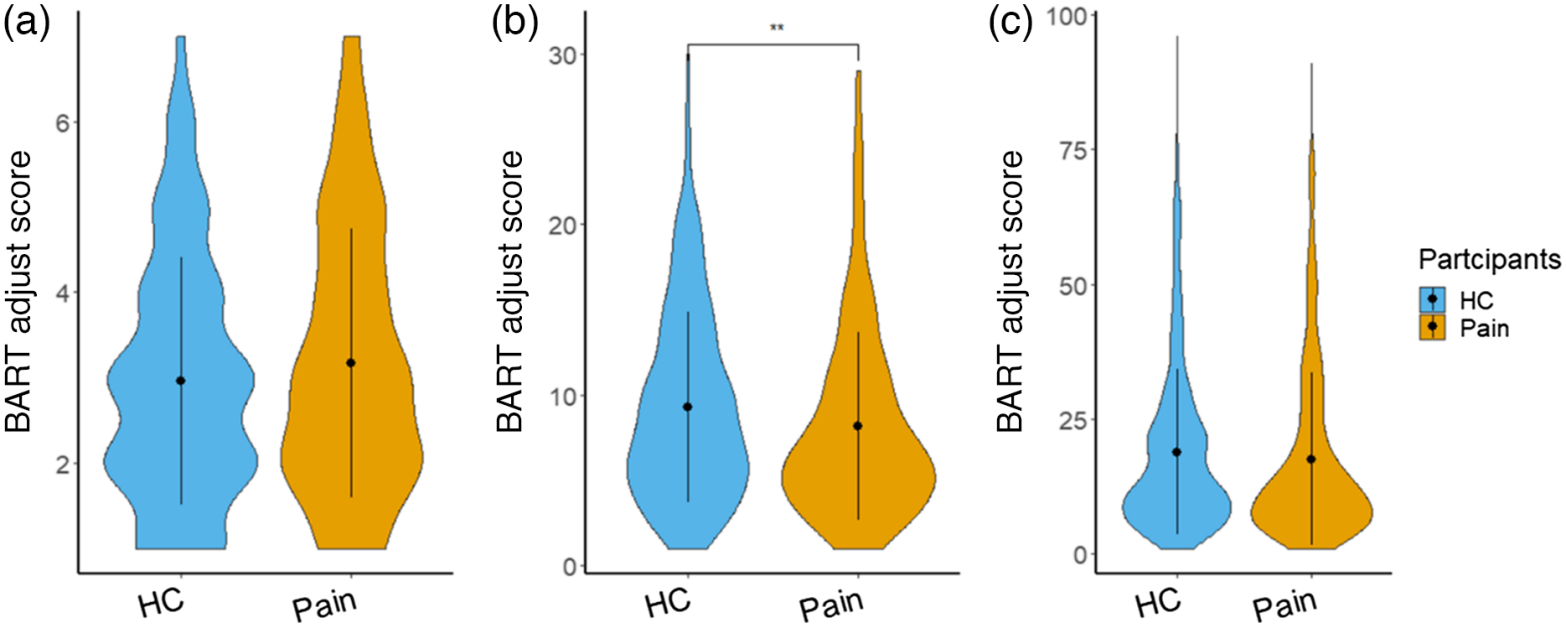
Adjusted BART scores across the three conditions. (a) Adjusted score for the blue balloon condition (low explosion threshold), (b) adjusted score for the green balloon condition (intermediate explosion threshold), and (c) adjusted score for the red balloon condition (high explosion threshold). * indicates p<0.05.

The chronic pain patient group exhibited significantly lower adjusted BART scores (7.89±3.00) when pumping the green balloons (intermediate explosion threshold condition) compared with the HCs (10.35±4.56), t(2,49)=−2.30, and p=0.026 [Fig. 3(b)]. In addition, for the other two conditions, the patient group showed no significant difference in adjusted BART scores. For the high explosion threshold condition (red balloons), the patient group showed slightly more conservative potentials (18.79±14.80) than the HCs (20.98±14.24), t(2,49)=−0.51, and p=0.609 [Fig. 3(c)]. However, this is not the case for the low explosion threshold condition (blue balloons), in which the patient group demonstrated a bit riskier potential (3.27±0.76) than the HCs (3.09±1.45), t(2,49)=0.53, and p=0.604 [Fig. 3(a)].

### 3.2 Computational Modeling

#### 3.2.1 Model comparison

Results from the RFPM and EWMVM models were compared using corresponding LOOIC weights that denote the relative likelihood defined by LOOIC values for selecting an optimal model. A low LOOIC value specifies a more fit model. Comparison results (Table 3) illustrated that EWMVM is a better fit for our results according to its LOOIC weights of 1.

**Table 3:** LOOIC results of the two models in the two groups.

Group	Model	LOOIC value	LOOIC weight
HC	EWMVM	3251.459	1.000
	RFPM	3298.085	0.000
Chronic pain	EWMVM	2137.184	1.000
	RFPM	2224.345	0.000

EWMVM, exponential-weight mean-variance model; RFPM, re-parameterized four-parameter model.

#### 3.2.2 Model parameters

**Fig. 4:**
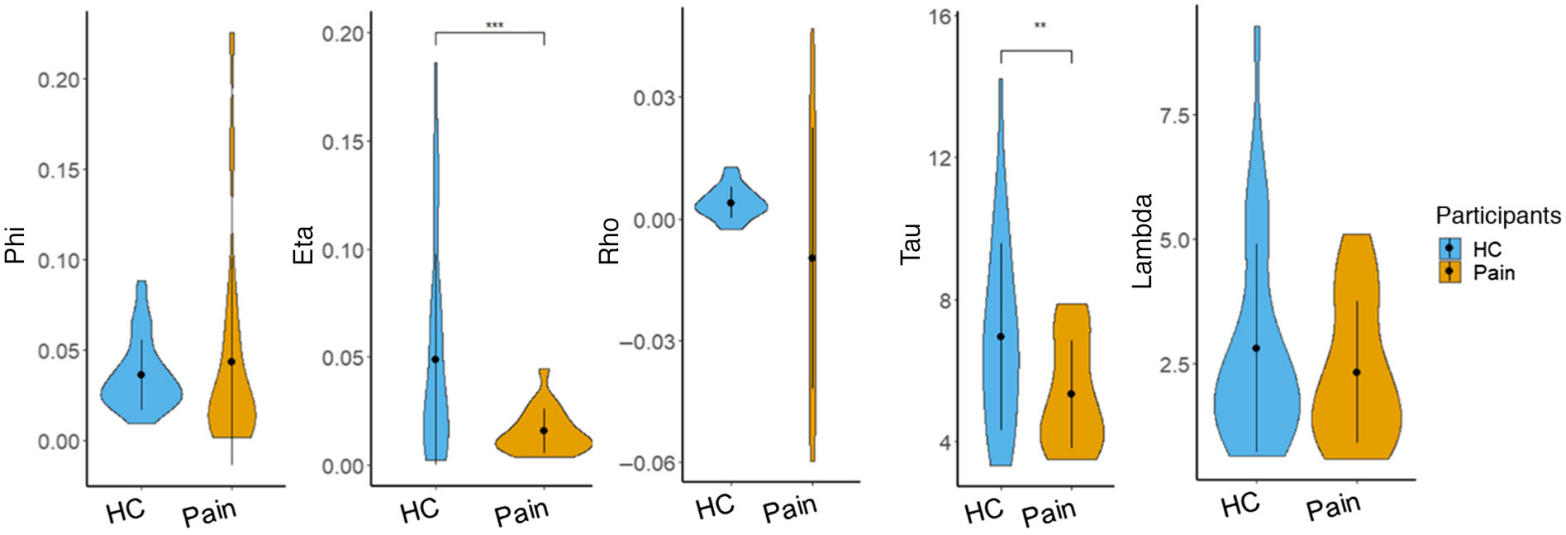
Group parameters with EWMVM. Phi is used to describe prior belief of burst, eta displays the updating exponent, rho denotes risk preference, tau represents inverse temperature, and lambda represents loss aversion. ** indicates p<0.01, and *** indicates p<0.001.

The posterior distributions of the fitted model (EWMVM) parameters were calculated to quantify describe the underlying cognitive process when the two groups carried out BART (Fig. 4). It was found that the chronic pain patients exhibited significantly decreased learning rates with η=0.0016, compared with HCs with η=0.049 (t(2,49)=−3.67,p=0.0008). Meanwhile, the chronic pain patients showed significantly lower τ values (τ=5.32) than HCs (τ=6.94) (t(2,49)=−2.78, p=0.007), indicating a low deterministic process when making risky decisions. By contrast, no significant difference was detected between the two groups in prior belief of the balloon not bursting ($\phi_{\text{pain}}=0.044$, $\phi_{\mathrm{HC}}=0.037$, t(2,49)=0.5584, and p=0.5822), risk-taking propensity ($\gamma_{\text{pain}}=-0.010$, $\gamma_{\mathrm{HC}}=0.004$, t(2,49)=1.910, and p=0.071), and loss aversion ($\lambda_{\text{pain}}=2.32$, $\lambda_{\mathrm{HC}}=2.807$, t(2,49)=−0.987, and p=0.3283). Interestingly, the low performances of BART and model parameters (learning rate η and inverse temperature τ) concurrently demonstrated the deficit in risky decision-making abilities among chronic pain patients.

### 3.3 fNIRS Neuroimaging Results

**Fig. 5:**
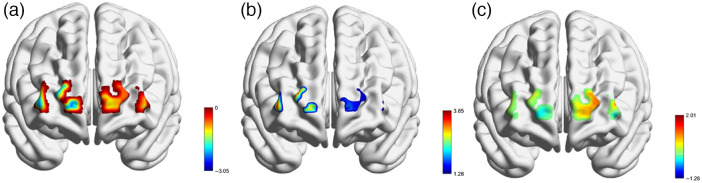
Activation patterns of the PFC in BART. (a) T-maps show the activation difference of the PFC during BART between the chronic pain group and HC group, (b) the activation patterns of PFC during BART for the HC group, and (c) the activation patterns of PFC during BART for the chronic pain group. The color bar denotes the t values.

To verify the corresponding neural response of BART, the brain activation regions in the PFC were identified by fNIRS neuroimaging. The pairwise t-test was performed, demonstrating that significantly lower activation with $p_{\mathrm{FDR}}<0.05$ was detected for the chronic pain patient group, compared with HCs in channel 4 (t(2,49)=−3.049, $p_{\text{uncorrect}}=0.0037$, 36.09% of the frontopolar area and 63.91% of the orbitofrontal area). The t values of HbO signal difference between the chronic pain patients and HCs were mapped (Fig. 5) by adopting the Xjview toolbox^44^ and BrainNet Viewer toolbox.^45^

## 4 Discussion

Consistent with previous findings,^17^^,^^46^^,^^47^ the defective decision-making abilities were identified for chronic pain patients according to both adjusted BART score and modeling parameters in our current study. Interestingly, adjusted BART scores were introduced by Lejuez et al.^21^ to measure the performance of risky decision making, which is indirectly affected by the explosion probability. This work also found that, compared with HCs, the patient group showed significantly decreased activation patterns of risky decision-making behaviors, which might be due to the chronic pain induced by physical damage or abnormal psychological feelings. In particular, cognitive functions were highly influenced by chronic pain, as cognitive activities might require resource allocation to subjectively perceive pain.^48^^,^^49^ In addition, previous studies also demonstrated that the impaired performance of various cognitive processes has been found during acute pain stimulus^30^^,^^48^^,^^50^ or chronic pain stage (suffering from long-lasting aberrant pain stimulus).^51^^–^^54^ In particular, pain perceptions compete with other cognitive functions, thus demanding attention by the limited cognitive resources.^55^ For example, Moore et al.^56^ found that chronic pain patients showed decreased attention allocation. Meanwhile, memory and storage systems of information, including working memory^57^ and recognition memory,^58^ were revealed to be negatively influenced by chronic pain. Further, as a complex cognitive process, decision making involves multiple cognitive functions. Therefore, impaired decision-making performance was detected in chronic pain for this work, highlighting the importance of identifying the core deficit in decision making associated with chronic pain.

Importantly, through constructing the computational model, we found that EWMVM^25^ is a better model as it took loss aversion of the participants into account. In EWMVM, the performance of BART is determined by five factors: prior belief of the balloon not bursting (ϕ), learning rates based on results of each trial (η), risk-taking propensity (γ), inverse temperature (τ), and loss aversion (λ). Our findings demonstrated significantly impaired learning abilities (update the belief after feedback) and more random decision making among chronic pain patients. Learning from feedback and updating the decision-making strategy to make optimal decisions are extremely important due to the uncertain characteristics of the outcome in decision making.^59^ When making decisions, participants need to adjust their decision-making strategy based on the feedback via two distinctive yet interactive patterns: “cold” cognitive pattern and “hot” emotional response.^60^ The former is related to the central executive network, anchored in dorsolateral PFC, which evaluates and optimizes decision-making strategy to the maximal profit.^61^ Using BART, Humphreys et al.^62^ identified that, with the development of brain from childhood to adulthood, the learning abilities increase gradually and become less likely to be affected by negative feedback. By contrast, for the “hot” emotional response,^63^ emotional and involuntary arouse occurred in body, demonstrating increased heart rate, visceral reactions, transpiration or relief, and regret when receiving feedback.^64^ Interestingly, both acute pain and chronic pain can be the dominant stressors to evoke the stress response system [the hypothalamic-pituitary-adrenal (HPA) axis], releasing the stress hormone cortisol. Importantly, those responses are remarkably similar to the response induced by stressful emotions.^65^ For example, numerous studies have illustrated the dysfunction of HPA in chronic pain patients, by abnormally releasing cortisol and biasing toward negative feedback.^66^^,^^67^ In addition, Mansour et al.^68^ proposed that pain can provide a teaching signal that enables individuals to reduce negative feedback in which pain is a primary punisher to give rise to negative reinforcement. Therefore, our evidence clearly indicated that impaired decision-making performance could be explained by decreased learning abilities from feedback. And it is more likely that chronic pain patients are willing to make decisions with deliberations, which might be largely influenced by pain feelings.

Further, using fNIRS neuroimaging techniques, we identified decreased cortical activation in the PFC during risky decision making for the chronic pain patient group, compared with HCs. The PFC has been recognized to play a vital role in perceiving, modulating, and reappraising pain through various ascending and descending tracts.^69^ Due to the long-lasting chronic pain stimulus, PFC has to allocate more resources to process pain-related information and strengthen pain-related pathways.^70^ Those pain-induced alternations would influence other functions of the PFC, including decision making. Meanwhile, the abnormal functions of PFC have been widely inspected in chronic pain patients, including working memory task,^71^ time discounting,^72^ and attention task.^73^ In particular, previous studies demonstrated significant structural and functional alterations of PFC. For example, reduced gray matter volume in PFC was detected in chronic pain patients, including musculoskeletal pain^74^^–^^77^ and other types of chronic pain.^78^^–^^80^ In addition, the altered structural and functional connectivity of PFC has been revealed for chronic pain patients. For example, Moayedi et al.^81^ demonstrated reduced white matter connectivity from the middle cingulate cortex to PFC, and Čeko et al.,^82^ Hashmi et al.,^83^ Hubbard et al.,^84^ and Li et al.^85^ detected abnormal functional connectivity of PFC.

The distinctive risky decision-making behaviors and deactivated PFC activity during risky decision making of chronic pain patients provide an effective index of automatically discerning the chronic pain patients from HCs and measuring the intervention efficacy. Several machine learning methods have achieved satisfactory performance in the diagnosis of chronic pain patients by combining cognitive tasks and neuroimage tools.^86^^,^^87^ Further studies could utilize artificial intelligence, such as machine learning and deep learning, to combine cognitive and neuroimaging data for precise diagnosis and treatment of chronic pain patients. However, there were several limitations in our study which warrant discussion. First, the channels of fNIRS in the current study were unable to cover the whole PFC, and dorsolateral PFC, frontopolar regions, and ventromedial PFC might play different roles in risky decision making. Thus, future studies can make more precise discriminations of those regions and non-PFC brain regions in chronic pain patients during risky decision making. Second, even though we excluded patients diagnosed with psychiatry disorders (anxiety, depression, etc.) and patients with extreme pain, our study did not measure the specific degree of anxiety, depression, pain intensity, and pain interference among the included sample, which could be taken into consideration by future investigations.

In summary, through constructing computational models to quantitatively depict the underlying process of BART, we demonstrated that chronic pain patients have significant deficits in learning from the BART results and make decisions more randomly without deliberation. In addition, the deactivated brain activation in PFC was manifested for the chronic pain patient group, supporting the notion that long-term aberrant pain stimulus might damage the functions of the PFC. Combined evidence from behavioral and neuroimaging findings suggest that the altered risky decision-making abilities can provide new insights for discerning pain-related cognition impairment and understanding the cognitive neural mechanism of pain-related circuits, further achieving better intervention efficacy.

## Code, Data, and Materials Availability

The code and datasets generated for this study are available on request to the corresponding author.
